# Supplementary material for: Statistical Evolutionary Laws in Music Styles
Source: Sci Rep. 2019 Nov 5;9:15993. doi: 10.1038/s41598-019-52380-6 (PMC6831699; doi:10.1038/s41598-019-52380-6)
Supplement: Supplementary file 1 — Supplemental Material [file 41598_2019_52380_MOESM1_ESM.pdf]

Supplemental Material for "Statistical Evolutionary Laws in Music Styles"

Eita Nakamura[1*] and Kunihiko Kaneko[2†]

[1] *The Hakubi Center for Advanced Research and Graduate School of Informatics, Kyoto University, Sakyo, Kyoto 606-8501, Japan*

[2] *Department of Basic Science, University of Tokyo, Meguro, Tokyo 153-8902, Japan*

Contents

1 Analysis on the Classical Music Data **1**

2 Analysis of the SCE models for other distributions **2**

3 Additional Comparison between the SCE Model and the Log-Potential Model **4**

1 Analysis on the Classical Music Data

We can analyze frequencies of non-diatonic motions in the same way as for frequencies of tritones (Fig. 1 in the main text). The result is shown in Fig. 1. We can find the same statistical tendencies that are found for the frequencies of tritones, even though they are less clear:

- Beta-like distribution of frequency features
- Steady increase of the mean and standard deviation
- Nearly constant ratio of the mean and standard deviation

We used the birth year of the composer as the reference time of each musical piece in Fig. 1 in the main text and Fig. 1 in this Supplemental Material. This is because the composition year for each individual piece is not given in the dataset used. Alternatively, if we use as the reference time the death year, the middle year (defined as the average of the birth and death years), and the active year (defined as the birth year plus 35 years to represent the active time of the composer's career) of the corresponding composer, we obtain similar results apart from shifts in time.

*Electronic address: eita.nakamura@i.kyoto-u.ac.jp

†Electronic address: kaneko@complex.c.u-tokyo.ac.jp

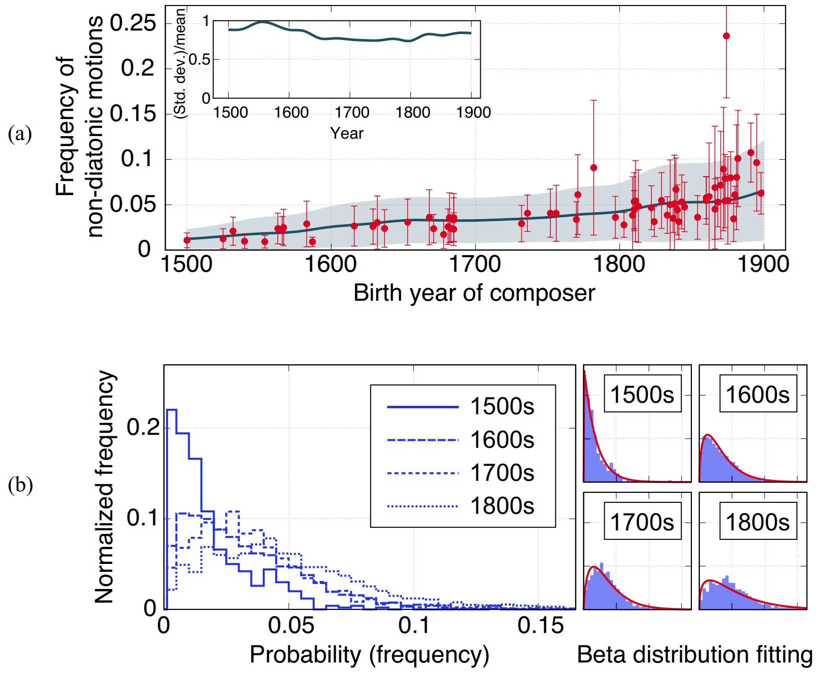

Figure 1: Evolution of the distribution of frequencies of non-diatonic motions observed in Western classical music data. In (a), points and bars indicate the mean and standard deviation for each composer, and the step line and shade indicate those for time windows of 100-year width shifted in units of 25 years (spline interpolation applied). In (b), distributions obtained for each century.

2 Analysis of the SCE models for other distributions

In the main text, we analyze the SCE model for the beta distribution. Here we analyze the SCE models defined with the gamma and log-normal distributions to show the generality of the model analysis result, especially the existence of a slow manifold when the novelty term is active. A gamma distribution is defined as

$$\phi_t(\theta) = \mathrm{Gamma}(\theta; a_t, b_t) = \frac{b_t^{-a_t}}{\Gamma(a_t)} x^{a_t-1} e^{-x/b_t}, \tag{1}$$

and the parameters $a_t, b_t > 0$ are related to the mean and standard deviation as

$$\mu_t = a_t b_t, \quad \sigma_t = b_t \sqrt{a_t}. \tag{2}$$

A log-normal distribution is defined as

$$\phi_t(\theta) = \mathrm{LN}(\theta; \tilde{\mu}_t, \tilde{\sigma}_t) = \frac{1}{\sqrt{2\pi}\tilde{\sigma}_t \theta} \exp\left[-\frac{(\ln\theta - \tilde{\mu}_t)^2}{2\tilde{\sigma}_t^2} \right], \tag{3}$$

and the parameters $\tilde{\mu}_t \in (0, \infty)$ and $\tilde{\sigma}_t > 0$ are related to the mean and standard deviation as

$$\mu_t = \exp(\tilde{\mu}_t + \tilde{\sigma}_t^2/2), \quad \sigma_t/\mu_t = \sqrt{\exp(\tilde{\sigma}_t^2) - 1}. \tag{4}$$

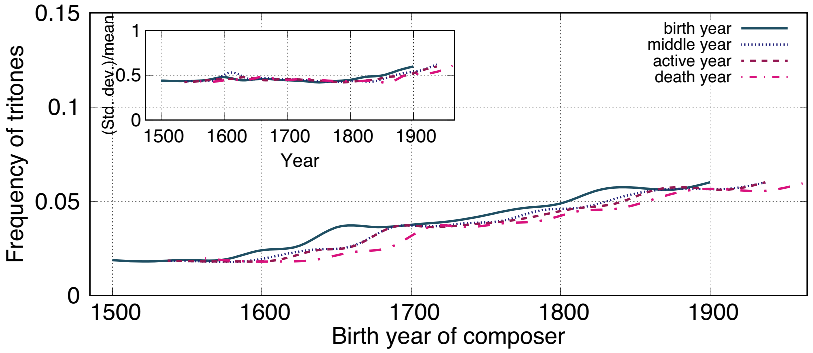

Figure 2: Effect of using different reference times for the Western classical music data. Evolutions of the mean and standard deviation of frequencies of tritones, corresponding to Fig. 1(a) in the main text, are illustrated.

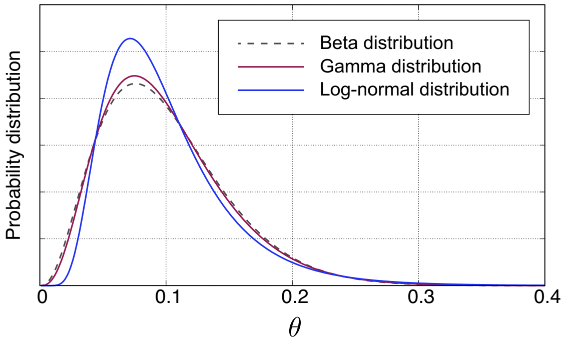

Figure 3: Beta, gamma, and log-normal distribution for the same mean and standard deviation ($\mu = 0.1$ and $\sigma = 0.05$).

Both of these probability distributions are defined in the range $\theta \in (0, \infty)$, so they are not strictly proper for the probability parameter θ restricted in the range $(0, 1)$. Nevertheless, when the mean μ_t is smaller than unity and the standard deviation is sufficiently small, the supports of these distributions are effectively bounded in the range $(0, 1)$. We study these distributions for the demonstration purpose.

The gamma, log-normal, and beta distributions are compared in Fig. 3, where distributions with the same mean and standard deviation ($\mu = 0.1$ and $\sigma = 0.05$) are shown. As we see in the figure, the shapes of these three distributions are generally similar for a small mean and for a standard deviation smaller than the mean.

SCE models for the gamma and log-normal distributions are defined by substituting Eqs. (1) and (3) into Eq. (4) in the main text, respectively. We can conduct numerical analyses similarly as in the main text. Focusing on the case $\beta_N > 0$ and $\beta_T = 0$, results of numerical analyses are shown in Figs. 4 and 5. From the results in the figures and with the same argument as in the main text, one can see a slow manifold in which σ_t/μ_t is kept almost constant and μ_t grows nearly exponentially, similarly as in the case of the beta distribution.

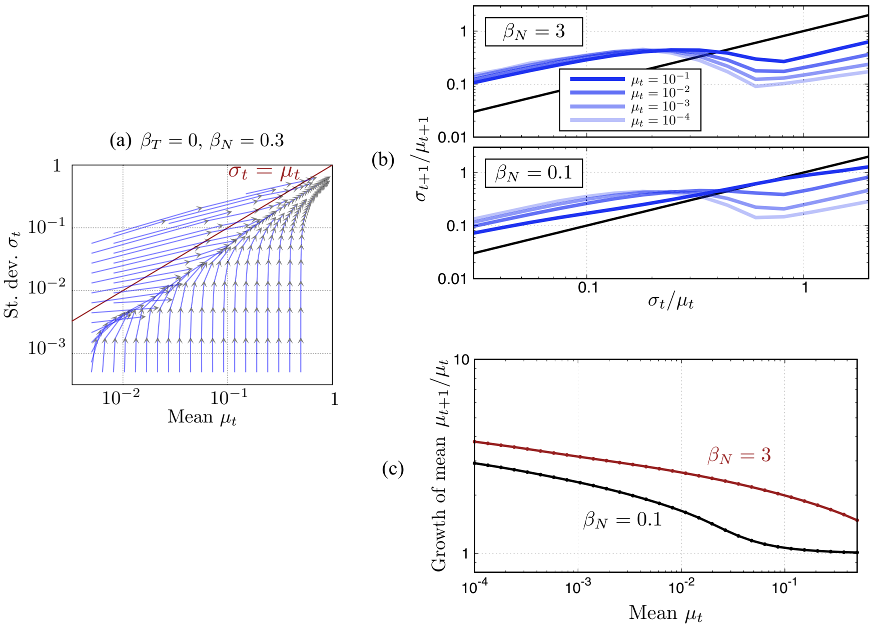

Figure 4: Numerical analysis of the SCE model defined with the gamma distribution for the case $\beta_N > 0$ and $\beta_T = 0$. (a) Orbits of the SCE model (b) Dynamics of the ratio σ_t/μ_t. (c) Growth of the mean around the slow manifold ($\sigma_t/\mu_t = 0.4$).

3 Additional Comparison between the SCE Model and the Log-Potential Model

In Figs. 4(a) and 4(b) of the main text, we compared how the SCE model and the log-potential model can fit the real data of classical music. There, the model parameters were optimized to best fit the two sets of data (frequencies of tritones and non-diatonic motions). Here we report the results when the model parameters are fitted to the two sets of data individually.

The results are shown in Figs. 6(a) and 6(b). The root mean squared errors of the (tritone, non-diatonic motion) data are (4.4×10^{-3}, 7.2×10^{-3}) for the SCE model and (3.0×10^{-3}, 5.8×10^{-3}) for the log-potential model. Compared to the results in the main text, these results show that for the SCE model the precision of the individual fit is similar to that of the simultaneous fit, and that the log-potential model can fit individual data slightly better than the SCE model. This shows that although the log-potential model is flexible for fitting individual sets of data, it cannot fit both sets of data simultaneously, confirming that it is not trivial to fit both sets of data in a unified manner.

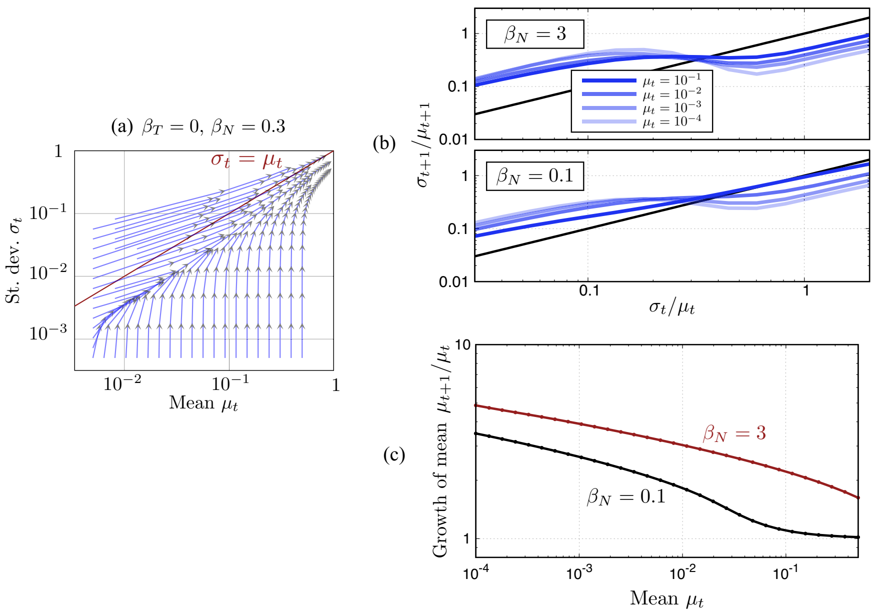

Figure 5: Numerical analysis of the SCE model defined with the log-normal distribution for the case $\beta_N > 0$ and $\beta_T = 0$. (a) Orbits of the SCE model (b) Dynamics of the ratio σ_t/μ_t. (c) Growth of the mean around the slow manifold ($\sigma_t/\mu_t = 0.4$).

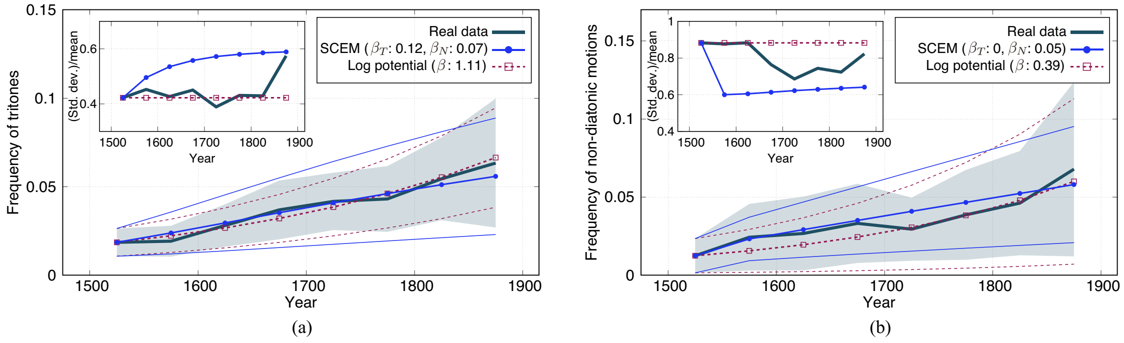

Figure 6: Comparisons between model predictions and real data. Bold lines indicate means, and thin lines and shadow indicate the ranges of ± 1 standard deviation. Model parameters are optimized individually to fit the two datasets to minimize the squared error of predicted means and standard deviations (optimal parameters are shown in the insets).
